# Modified Cervicofacial Flap for Large Cheek Defect Reconstruction under Local Anesthesia

**DOI:** 10.1155/2019/1960609

**Published:** 2019-04-04

**Authors:** Shiv Prasad Sharma, Akshay Nigam

**Affiliations:** ^1^Zulfi General Hospital, Ministry of Health, Saudi Arabia; ^2^Radiation Oncology, G.R. Medical College, Gwalior, India

## Abstract

The cervicofacial flap was first described in 1969. For the past several years, it has been the flap of choice for the reconstruction of facial defects especially cheek defects. In recent years, with the advent of microvascular free flap tissue transfer, the use of a cervicofacial flap has been sparse. This article highlights the importance and application of locoregional flaps such as a cervicofacial flap in the reconstruction of soft tissue defects on a face. This case was unique from the reconstruction point of view. An 81-year-old patient presented to us with a long standing ulcerated growth in his left cheek which was neglected due to lack of symptoms and his poor financial condition. Clinically, the extent was defined from the suborbital skin region till the mid cheek region and medially from the lateral nasal crease up to the cheek prominence area. An initial biopsy suggested basal cell carcinoma. The lesion was excised, and a 4.5 × 5.5 cm defect was reconstructed using a modified “cervicofacial” flap under local anesthesia. Our experience shows that this technique is a reliable, easy to harvest flap with good outcomes. Moreover, it can be utilized in those candidates not suitable for a free flap procedure under general anesthesia.

## 1. Introduction

Reconstruction of large cutaneous defects involving a face poses a great surgical challenge. The microvascular free tissue transfer techniques are used primarily for the repair of extensive composite cheek defects. Those candidates, who are not suitable for such prolonged operations under general anesthesia, can be managed using locoregional flaps such as cervicofacial flaps with favorable outcomes. The first report of various local flaps for cheek reconstruction was by Esser et al. [1]. Mustarde utilized different cervical rotation flaps for the repair of orbital defects [2, 3]. This flap technique has evolved since its introduction, and several modifications have been published in the literature. Basically, two types of cervicofacial flaps have been described based on the plane of subcutaneous elevation and deep plane.

Over the past 2-3 decades, the reporting of these flaps has been scanty. Most studies published are retrospective in nature. We utilized a modified form of a cervicofacial flap (inferiorly based) for the reconstruction of a large Zone 1 cheek defect in our case [4]. This flap is based on robust random subcutaneous vascularity. The entire procedure in our case was performed under local anesthesia affirming that it is a simple, reliable, and easy to harvest technique which should be used in suitable patients. Our technique reduced the operating time and postop recovery time considerably. The final outcome achieved is comparable to any standard procedure. This case highlights the fact that even large defects can be treated under local anesthesia with this flap.

## 2. Case Presentation

**Figure 1:**
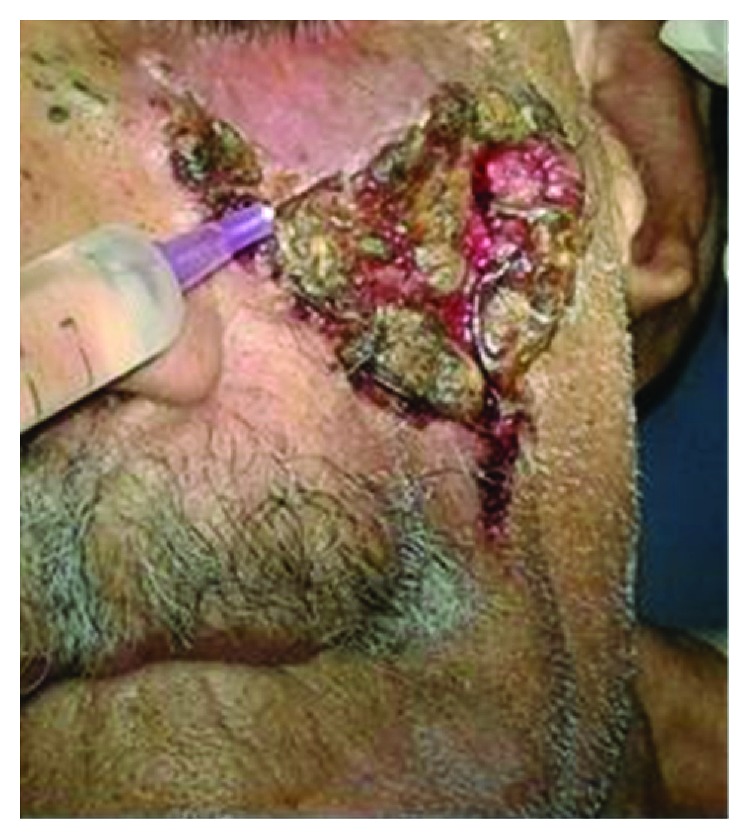
Ulcerated lesion involving the left cheek.

An 81-year-old male presented with a long standing ulcerated growth involving his left side face (Figure 1). His comorbidities included diabetes and hypertension. He had asymptomatic inguinal hernia. The initial histopathological diagnosis confirmed basal cell carcinoma. The lesion did not invade the underlying bone. Based on the diagnosis and the general condition of the patient, it was planned to perform wide excision+reconstruction with a modified cervicofacial flap. The entire procedure was performed under local anesthesia as the patient was a high risk category for general anesthesia.

## 3. Procedure

**Figure 2:**
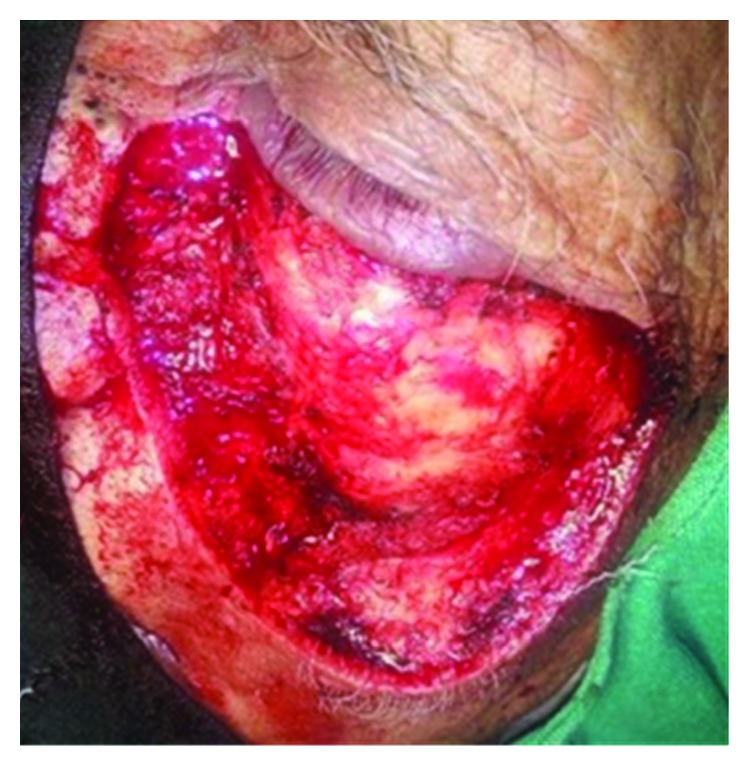
Defect following tumor resection.

**Figure 3:**
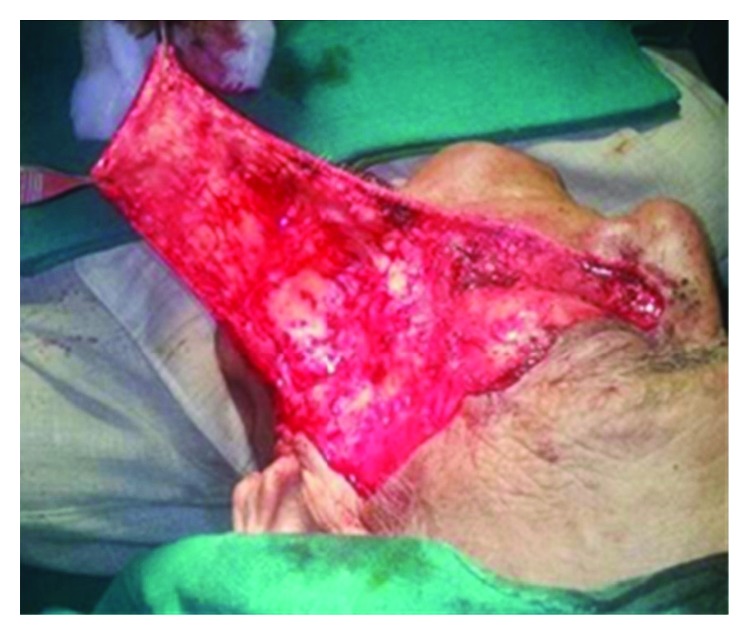
Elevated flap.

**Figure 4:**
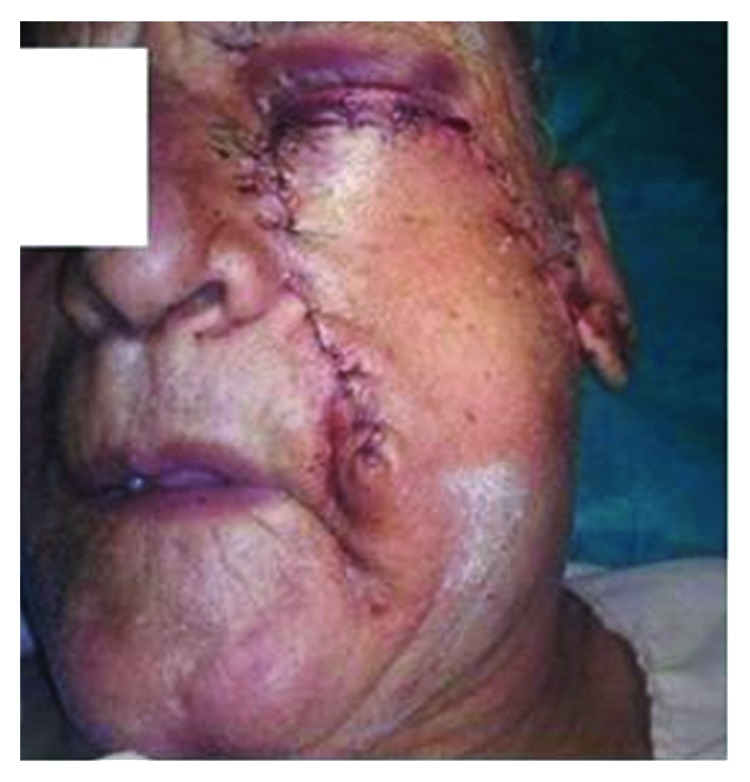
Immediate post op. Simultaneous primary closure of the donor site.

**Figure 5:**
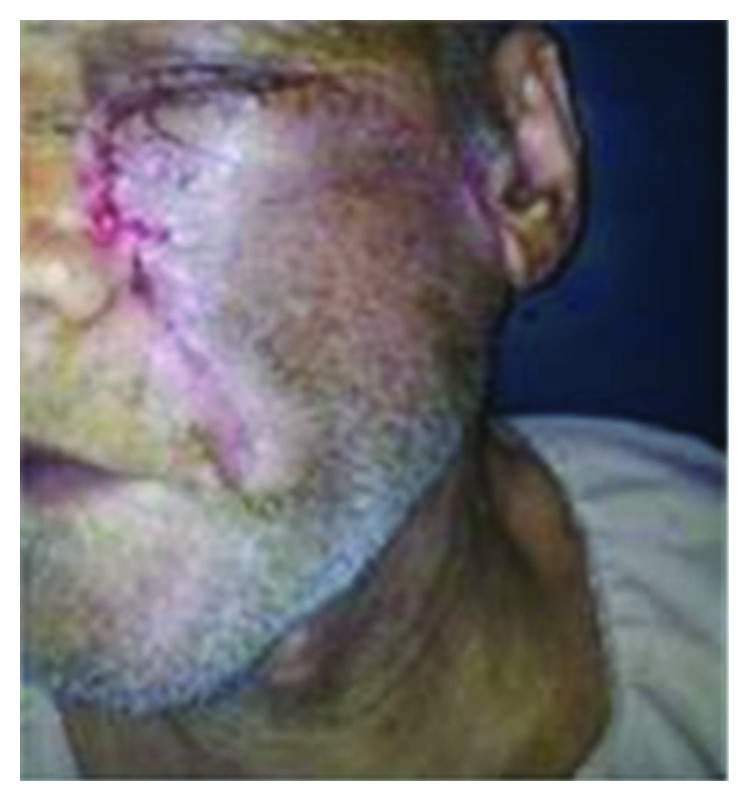
Follow-up showing good flap take-up and esthetics.

Wide excision of the lesion with a 5 mm margin was done (Figure 2). The incision for the flap started directly posterior from the superior most extent of the resection and carried straight till the preauricular crease for improved cosmetics. Medially, the incision was made without disturbing the anatomic boundary of the nose. The entire flap was elevated in a subcutaneous plane till along the lateral aspect of the mandible to enhance the reach and rotation of the flap (Figure 3). The entire defect could be reconstructed with good esthetic and functional results (Figure 4). This technique reduced the operating and recovery time without compromising the results. The postop period was uneventful with no observation of flap necrosis or significant facial nerve deficits. There was no other unanticipated outcome when the patient followed up for suture removal (Figure 5).

The flap design was straightforward with slight modification to a conventional cervicofacial flap. Even though deep plane dissection has been advocated by some to improve vascularity and reduce complications, it has been shown that the subcutaneous flap is much easier to raise with comparable and even lower complication rates than deep plane flaps [5, 6]. Also, performing a deep plane dissection needs more surgical expertise and experience. The reconstruction was one stage and did not require any later refinements.

## 4. Discussion

Reconstruction of cheek defects is a complex procedure and needs a lot of consideration with respect to esthetic and functionality and respecting the adjacent anatomic subunits. A useful classification of cheek defects was given by Roth et al. Based on the location of the defect, it can be categorized into three zones. Zone 1 refers to suborbital defects, Zone 2 includes defects in preauricular/temporal areas, and Zone 3 is the perioral, lower cheek and lateral mandibular region. A straightforward algorithm for this flap design was recently published by Al Shetawi et al. in a series of cases [7].

Rapstine et al. conducted a retrospective review of over 400 cases of cheek reconstruction [8]. Based on size, reconstructive options can vary from a simple primary closure, skin grafting, local advancement/rotation flaps, locoregional flap, and free flap. In the case of smaller cheek defects, surrounding tissues can be undermined to facilitate a primary closure. It can be practical in defects up to 4 cm [9]. But due consideration should be given to the adjacent anatomy, and distortion should be minimized. Skin grafting is a simple and effective way to cover cheek defects but with poor outcomes in terms of skin color and thickness match. Currently, the only possible scenario to utilize skin grafts for cheek repair is in the case of a severe medically compromised patient unfit for complex surgery under prolonged anesthesia. Free flaps such as radial forearm free flaps and anterolateral thigh flaps are usually reserved for very large composite defects in patients fit for prolonged surgery. Also, a free flap technique needs more surgical expertise; most of the time, the flap seems too bulky compromising exact skin color and texture match. Another difficulty in the free flap technique is the need for further refining procedures to improve outcomes.

Local flaps such as transposition, advancement, rotation, and nasolabial flaps can be the technique of choice in most cases of small to moderate cheek defects. For large cheek defects, distant flaps such as cervicofacial, cervicopectoral, deltopectoral, and pectoralis major myocutaneous flaps are excellent choices. The cervicofacial flap is more superior in terms of the ease of elevation, excellent skin color, and texture match, and also, the donor site can be closed simultaneously.

Based on the plane of elevation, subcutaneous and deep plane cervicofacial flaps exist [10, 11]. The former depends on the rich subdermal plexus of vessels, and the later relies upon more reliable large perforator branches from facial and transverse facial arteries [12]. Commonly encountered complications with these flaps are distal flap edge necrosis, lower eyelid ectropion, and hematoma [13, 14]. The deep plane technique has reduced the rate of these postop sequelae. In our case, we did not observe any of these findings till our last follow-up. However, we understand that more follow-up is necessary to identify late effects such as ectropion.

## 5. Conclusion

The cervicofacial flap is a versatile technique with excellent vascularity and good esthetic outcome which should be utilized in the reconstruction of facial defects. However, associated complications must be kept in mind and the patient should be well informed. The long-term follow-up of the patient is important to detect late complications such as ectropion. The field of reconstruction is constantly evolving with the integration of multiple disciplines. Advancements such as virtual surgical planning and 3D printing and role of stem cells might change our perspective in the future. But time-tested local flaps should always be a part of a surgeon's armamentarium.
